# Short-term effects of urban versus forest bathing on stress, mood, sleep, immune function, and metabolic health: a randomized, open-label, 2 × 2 crossover trial

**DOI:** 10.1265/ehpm.26-00092

**Published:** 2026-08-27

**Authors:** Xiangheng Dai, Dongteng Liao, Jianghua Ouyang, Hongwei Xiao, Zhongjie Tian, Liujin Qiu, Jinmei Yang, Hao Wang, Daocheng Gong, Zhaojia Li, Xu Wang, Yunsong Xu, Xiang Fang, Jianmin Tan, Meixiang Huang, Beidi Zhou, Huagui Peng, Yihua Peng, Qiang Wu

**Affiliations:** 1 Department of Science and Education, Shaoguan First People’s Hospital, Affiliated Hospital of Shaoguan University, Shaoguan, China; 2 Tianjingshan Forest Farm of Guangdong Province (Guangdong Tianjingshan National Forest Park Management Office), Shaoguan, China; 3 The First Clinical College of Guangdong Medical University, Zhanjiang, China; 4 Department of Respiratory Medicine, Shaoguan First People’s Hospital, Affiliated Hospital of Shaoguan University, Shaoguan, China; 5 College of Environment and Climate, Jinan University, Guangzhou, China; 6 Guangdong Provincial Observation and Research Station for Atmospheric Environment and Carbon Neutrality in Nanling Forests, China; 7 Research Institute of Tropical Forestry, Chinese Academy of Forestry, Guangzhou, China; 8 Beijiangyuan National Forest Ecosystem Research Station, Nanling Mts. China, Guangzhou, China; 9 Princess Máxima Center for Pediatric Oncology, Utrecht University, Netherlands; 10 Department of Prevention and Health Care, Shaoguan First People’s Hospital, Affiliated Hospital of Shaoguan University, Shaoguan, China

**Keywords:** Forest bathing, Stress reduction, Mood enhancement, Sleep quality, Immune modulation, Metabolic health

## Abstract

**Background:**

Evidence regarding the effects of forest bathing on young adults, particularly in low-latitude regions, remains limited. A more comprehensive understanding of its psychological and physiological impacts is needed to inform its potential role in public health.

**Objectives:**

To compare the short-term effects of forest versus urban exposure on stress, mood, sleep quality, immune-related parameters, and metabolic markers in healthy young adults.

**Methods:**

In this randomized, open-label, 2 × 2 crossover trial, 36 participants were assigned to forest–urban or urban–forest sequences with a 1-week washout period. Participants walked 2.5 km twice daily in each environment. Psychological scales and fasting blood samples were collected before and after each intervention. Linear mixed-effects models accounting for period and sequence effects were applied.

**Results:**

Forest bathing significantly reduced perceived stress (PSS: 22.3 to 20.7) and mood disturbance (POMS: 104.6 to 93.2), and improved sleep quality (SRSS: 20.5 to 18.0) and insomnia severity (AIS: 6.2 to 4.8), whereas no comparable improvements were observed following urban exposure. Hematological analyses revealed modest reductions in total white blood cell count and selected leukocyte subsets after forest bathing, with values remaining within physiological ranges. Forest exposure was also associated with small decreases in fasting blood glucose and log-transformed triglycerides. In contrast, DHEA-S levels increased following urban exposure but remained stable during forest exposure.

**Conclusions:**

In healthy young adults, short-term forest bathing was associated with robust improvements in psychological well-being and sleep quality, accompanied by modest physiological modulation compared with urban exposure. These findings support the potential role of forest environments as a complementary strategy for stress reduction, while the clinical significance of short-term biological changes warrants further investigation.

**Clinical trial registration:**

This study was registered at the Chinese Clinical Trial Registry (Registration number: ChiCTR2500096972)

**Supplementary information:**

The online version contains supplementary material available at https://doi.org/10.1265/ehpm.26-00092.

## Introduction

Cardiovascular diseases (CVD) and dyslipidemia impose substantial economic and public health burdens worldwide, contributing to escalating healthcare costs and productivity losses [1]. Dyslipidemia, characterized by abnormal lipid levels such as elevated cholesterol and triglycerides, is a major contributor to atherosclerosis and cardiovascular events.

Epidemiological evidence indicates that both CVD and dyslipidemia are increasingly prevalent among younger populations [1, 2]. Data from the National Health and Nutrition Examination Survey (NHANES, 2017–2020) showed that the age-adjusted prevalence of hypertension among U.S. adults aged ≥20 years reached 46.7%. In China, a large cross-sectional study involving 75,880 adults reported a diabetes prevalence of 11.2%, a key metabolic disorder closely associated with hypertension [3]. Moreover, approximately 40% of young adults aged 20–39 exhibit dyslipidemia, with prevalence rising markedly over the past two decades [4]. These trends underscore the urgent need for early preventive strategies targeting metabolic and cardiovascular risk factors in young populations.

Sedentary behavior and insufficient physical activity are recognized contributors to CVD and dyslipidemia. Prolonged sitting has been associated with increased cardiovascular risk, independent of overall physical activity, whereas regular physical activity is linked to improved lipid profiles and cardiovascular health [5–11]. However, technological advances and urbanization have fostered increasingly sedentary lifestyles among young individuals [12].

Sleep disturbances have also emerged as important risk factors for cardiovascular health and lipid metabolism [13, 14]. Poor sleep quality and sleep disorders are associated with elevated blood pressure, inflammation, and adverse lipid profiles [15–18]. A meta-analysis of hypertensive patients in China reported a sleep disturbance prevalence of 52.5%, significantly higher than that in healthy controls [19]. Notably, sleep problems are increasingly common among young adults, with nearly one-third reporting insufficient sleep or sleep-related complaints [20]. This further complicates the landscape of cardiovascular risk factors, necessitating comprehensive approaches to intervention.

Environmental stressors, including air pollution and noise exposure, further contribute to cardiovascular and metabolic dysfunction. Exposure to air pollution has been linked to increased blood pressure and unfavorable lipid profiles [11, 21], while chronic noise exposure adversely affects cardiovascular health [22, 23]. Within this multifactorial risk context, forest bathing has emerged as a promising early intervention strategy [24, 25]. Originating in Japan, forest bathing involves immersion in natural forest environments and has been associated with multiple physical and psychological health benefits [26].

Recent studies suggest that forest bathing may improve cardiovascular parameters, lipid profiles, and sleep quality [26–31]. However, evidence remains limited regarding its effects on younger populations, particularly in low-latitude regions. Addressing this gap is essential for developing age-appropriate and context-specific preventive strategies.

Therefore, this study aims to investigate the effects of forest bathing on cardiovascular parameters, lipid profiles, sleep quality, psychological stress, and immune-related indicators in a young population, with the goal of providing evidence to support forest bathing as a feasible public health intervention for early cardiovascular risk reduction.

## Methods

### Study design and registration

This study was a single-center, randomized, open-label, 2 × 2 crossover trial with two intervention sequences (AB: forest-urban; BA: urban-forest). The protocol was prospectively registered at the China Clinical Trial Registration Center (ChiCTR2500096972) on February 10, 2025, with the pre-specified primary outcome being change in total scores of psychological scales. The trial comprised: 1. Intervention Phase 1: 5 kilometers walking in assigned environment (forest/urban); 2. Washout Phase: 7-day period to eliminate carryover effects; 3. Intervention Phase 2: Cross-over to alternative environment.

### Participant recruitment

Participants were 36 young healthy subjects recruited from June 2024 to July 2024 through WeChat official account advertisement of Shaoguan First People’s Hospital. About WeChat official account advertising link can be found at: https://mp.weixin.qq.com/s/MnY0GYHERw64mRRn625mIg. Participants were recruited based on the maximum capacity of the study funding and location. Participants first undergo qualification screening by filling out an online questionnaire, and potential qualified individuals are evaluated in detail face-to-face by Xiangheng Dai and Dongteng Liao to determine their qualifications. Participants who met the criteria were randomly assigned to equal AB or BA sequence groups via SPSS 22.0 statistical software, with Dongteng Liao performing the randomization using a random number of 200,000.

Participants were enrolled under the same health-screening rules we recently described in detail [32]. In brief, volunteers had to be 18–45 years old, never-smokers, with BMI 19–26 kg/m^2^ and body weight ≥50 kg (men) or ≥45 kg (women). The key exclusion categories were (1) active or chronic medical conditions judged clinically relevant by the investigator, (2) any condition or behaviour that could alter drug absorption, distribution, metabolism or elimination (recent surgery, special diets, concomitant medicines, excessive alcohol/caffeine, etc.), and (3) pregnancy, lactation or plans for pregnancy during the study. The full checklist is reproduced in Supplementary Table S1 for transparency.

### Intervention protocol

**Fig. 1:**
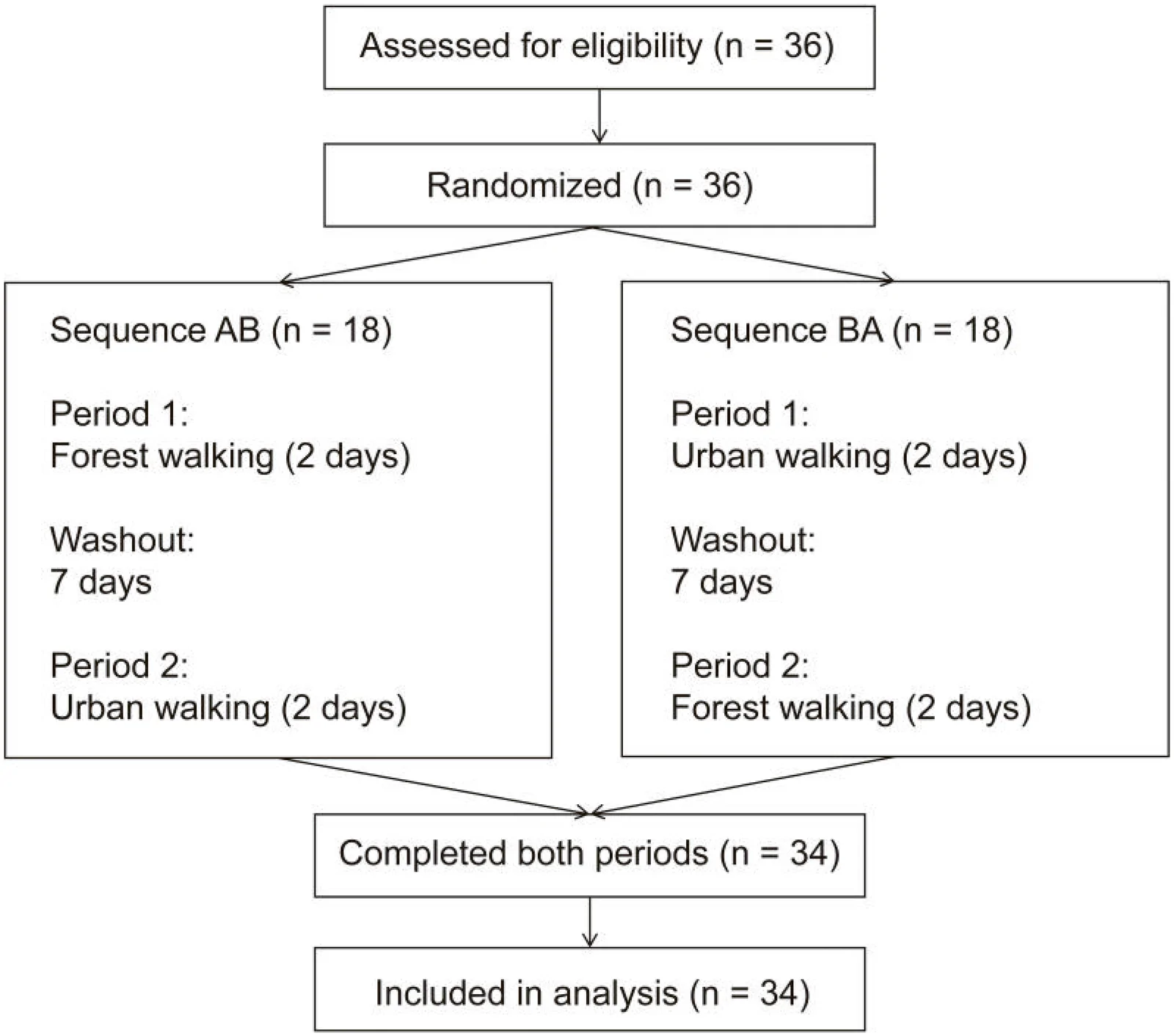
Study Design and Flowchart

The steps of the experiment are shown in Fig. 1. The first week of the experiment: Participants underwent baseline fasting blood sampling and completed questionnaires one day before each intervention phase. At 9:00 a.m. on the first day of the experiment, participants in group A walked 2.5 kilometers along the recreational trails in the forest park to experience forest bathing activities. Group B participants walked the same distance along the same forest bathing method in the urban section of Zhenjiang District, Shaoguan City. At 15:00 pm, both groups repeated the morning walking routine again, maintaining the same intensity and method of exercise. On the second day of the experiment, participants were required to undergo another blood test on an empty stomach in the morning and complete the corresponding questionnaire. Washout period: To effectively eliminate the potential effects of the previous phase of the experiment, a 1-week washout period was set up, during which the participants resumed their normal life rhythm and did not participate in any special intervention activities. The second week of the experiment: On the day before the start of the experiment, participants in both groups completed baseline measurements of the relevant indicators. In contrast to the first week, group A traveled to the city road, and group B traveled to the forest park recreation trail, and both groups completed the walk and related index measurements in the same way as in the first week. Participants reported no extreme exercise patterns, and none engaged in professional athletic training. Habitual physical activity levels were comparable between groups at baseline.

### Experimental location

The forest bathing site is located in the Tianjingshan Forest Farm, Ruyuan County, Guangdong Province. It lies in the western part of Ruyuan Yao Autonomous County, at the south foot of Wuling, a tributary of the Nanling Mountains (112°53′-113°15′E, 24°39′-24°51′N). The forest coverage rate is 96.7%. The region is characterized by a mountainous climate under the subtropical monsoon climate regime, with abundant rainfall and distinct dry and wet seasons. The average annual temperature ranges from 17 to 20 °C, and the daily relative humidity ranged from 75% to 95%. The zonal vegetation is dominated by evergreen broad-leaved forests.

The city bathing site is located at Walking Street Road, Zhenjiang District, Shaoguan City (24°48′5.0′′N, 113°36′5.0′′E). The average annual temperature ranges from 18.8 to 21.6 °C, and the daily relative humidity ranges from 70% to 90%.

### Measures

#### Questionnaire evaluation

As in our previous research [32], psychological stress, mood state, and sleep status were assessed using the Perceived Stress Scale (PSS), Profile of Mood States (POMS), Self-Rating Scale of Sleep (SRSS), and Athens Insomnia Scale (AIS).

The PSS, developed by Cohen et al. in 1983 [33], consists of 14 items assessing perceived stress during the past month. Each item is rated on a 5-point Likert scale ranging from 0 (never) to 4 (very often), yielding a total score from 0 to 56.

The POMS, originally developed by McNair et al. in 1965 [34], includes 65 adjectives describing different mood states, rated on a 5-point scale from 0 (almost none) to 4 (very much). The total mood disturbance score is calculated according to the standard scoring method by combining positive and negative subscales and adding a constant of 100.

The SRSS is a widely used instrument for subjective sleep assessment [35]. It consists of 10 items, each scored on a 5-point scale from 1 to 5, with higher total scores indicating poorer sleep quality.

The AIS, developed in 2000 by a research team in Athens, Greece [36], is an internationally validated self-report scale for insomnia assessment. The AIS includes 8 items, each rated from 0 (no problem) to 3 (serious problem), with a total score ranging from 0 to 24.

#### Routine blood test

Fasting venous blood samples were collected at multiple time points to assess short-term changes in routine blood parameters. Blood routine indices included white blood cell count and differential counts (neutrophils, lymphocytes, monocytes, eosinophils, basophils) [37–39], red blood cell count, hemoglobin concentration [40], and platelet-related parameters, including platelet distribution width [41].

#### Lipid profiles

Fasting blood samples were analyzed for lipid-related parameters, including total cholesterol (TC), triglycerides, low-density lipoprotein cholesterol (LDL-C), high-density lipoprotein cholesterol (HDL-C), non-HDL cholesterol [42], apolipoprotein A1 (ApoA1), apolipoprotein B (ApoB), and lipoprotein(a) [Lp(a)] [43, 44].

#### Blood glucose and hormonal responses

Blood glucose levels were measured dynamically during the study period. In addition, circulating concentrations of insulin, B-type natriuretic peptide (BNP), and dehydroepiandrosterone sulfate (DHEA-S) were assessed to evaluate hormonal changes associated with metabolic and physiological regulation.

### Statistical analysis

Statistical analysis was performed using GraphPad Prism software. First, normality tests (such as the Shapiro-Wilk test) were conducted to determine whether the data followed a normal distribution. For count data that were normally distributed, a mixed-effects model was used for analysis, with a Geisser-Greenhouse correction applied to adjust for violations of sphericity. Post-hoc pairwise comparisons were performed using the Holm-Sidák method. For count data that were not normally distributed, the Friedman test was employed. If the results were significant, pairwise comparisons were further conducted using Dunn’s multiple comparisons test; if the results were not significant, a log transformation was applied and normality was tested again. If the transformed data followed a normal distribution, a mixed-effects model was used for analysis, with a Geisser-Greenhouse correction applied to adjust for violations of sphericity, and post-hoc pairwise comparisons were performed using the Holm-Sidák method. If the transformed data still did not follow a normal distribution, the results of the original Friedman test were reported. A significance level of α = 0.05 was set, and differences with P values less than 0.05 were considered statistically significant. Following recommendations for crossover trials (Chen, 2018) [45], we performed:

- Sequence effect analysis using ANOVA on period differences;
- Period effect adjustment through inclusion of period as a fixed factor;
- Carryover effect evaluation via intervention-by-period interaction terms.

The linear mixed-effects model was specified as:

Y_ijk=μ+α_i+β_j+γ_k+π_l+ε_ijk

Where:

- α_i: random intercept for participant i
- β_j: fixed effect for period j (1 or 2)
- γ_k: fixed effect for intervention k (forest/urban)
- π_l: sequence group l (AB/BA)
- ε_ijk: residual error

Carryover effects were tested by including intervention × period interaction terms.

## Results

### Demographic information of subjects

Between June and July 2024, a total of 36 participants were randomized into a 2 × 2 crossover trial. One participant withdrew prior to trial initiation and one withdrew after completing the first intervention phase; both withdrawals occurred in Group B (see Adverse Events section). All remaining participants completed the study protocol as planned, and data for all primary and secondary outcome measures were complete.

Baseline demographic characteristics and clinical, psychological, hematological, metabolic, and hormonal parameters were comparable between Group A and Group B. No statistically significant differences were observed between the two groups for any baseline variables (all *P* > 0.05; Supplementary Table S2), indicating good baseline balance prior to intervention.

### Validation of 1-week washout period validity

To minimize potential carryover effects in this randomized, open-label, 2 × 2 crossover trial, a 1-week washout period was implemented between intervention phases. The adequacy of the washout period was evaluated using paired-sample t-tests to compare baseline measurements before Period 1 and Period 2, as well as mixed-effects models to assess Period × Sequence interactions.

Paired t-test analyses showed that most psychological, hematological, metabolic, and hormonal parameters did not differ significantly between baseline measurements across periods (all *P* > 0.05), although a limited number of variables exhibited statistically significant differences (Supplementary Table S3). To further assess potential carryover effects, mixed-effects models incorporating participant-level random effects were applied. No significant Period × Sequence interactions were observed for the majority of outcome variables (all *P* > 0.05), indicating the absence of systematic carryover effects between intervention phases (Supplementary Table S4). Taken together, these findings support the adequacy of the 7-day washout period in effectively minimizing residual effects from the preceding intervention phase.

### Effects of urban and forest bathing on psychological well-being

In this study, we evaluated the effects of urban and forest bathing on participants’ psychological well-being. By analyzing the changes in various scales, we found that forest bathing had a significant positive impact on psychological health.

**Fig. 2:**
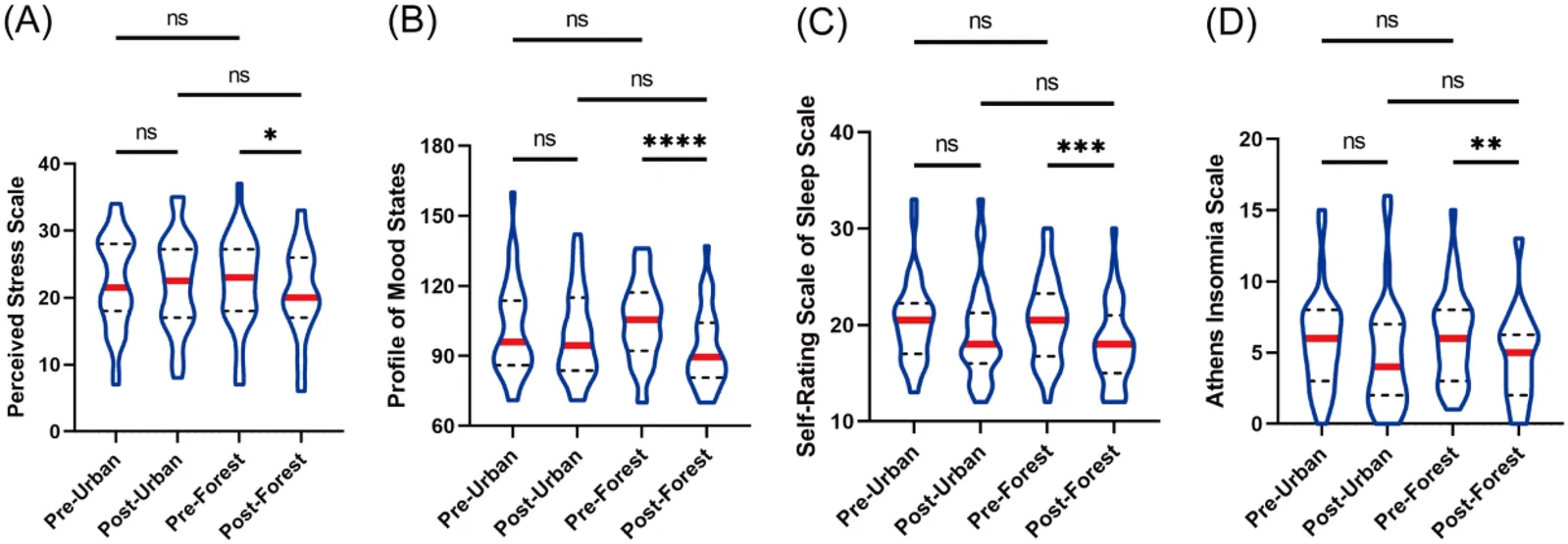
Effects of urban and forest bathing on psychological well-being. (A) Forest bathing significantly reduced perceived stress, with PSS scores dropping significantly from 22.3 to 20.7. (B) Forest bathing produced a substantial improvement, with POMS scores dropping significantly from 104.6 to 93.2. (C) Forest bathing markedly improved sleep quality, as evidenced by a significant drop in the SRSS scores from 20.5 to 18.0. (D) Forest bathing significantly reduced insomnia severity, with AIS scores falling from 6.2 to 4.8. Violin plots show individual values (blue), group medians (red dashed lines), and interquartile ranges (black dashed lines) before (“Pre-”) and after (“Post-”) each intervention. *P < 0.05, **P < 0.01, ***P < 0.001, ****P < 0.0001; ns, not significant.

First, we used the PSS to assess participants’ stress level (Fig. 2A). There were no significant changes in stress scores before and after urban bathing. However, after forest bathing, the average stress score significantly decreased from 22.3 to 20.7 (P < 0.05), indicating that forest bathing effectively reduced perceived stress.

Next, we assessed participants’ mood changes using POMS (Fig. 2B). Mood scores remained largely unchanged after urban bathing. In contrast, after forest bathing, participants’ mood scores improved significantly, decreasing from 104.6 to 93.2 (P < 0.0001). This suggests that forest bathing has a notable positive effect on enhancing mood.

Regarding sleep quality, we used SRSS (Fig. 2C), where lower scores indicate better sleep quality. Urban bathing did not significantly affect sleep quality, but forest bathing significantly improved it, with the average score decreasing from 20.5 to 18.0 (P < 0.001), highlighting the beneficial effect of forest bathing on sleep.

Finally, we assessed insomnia severity using AIS (Fig. 2D). There were no significant changes in insomnia scores following urban bathing. However, after forest bathing, participants experienced a significant reduction in insomnia severity, with scores decreasing from 6.2 to 4.8 (P < 0.01), indicating that forest bathing alleviated insomnia symptoms.

In conclusion, while urban bathing had limited effects on psychological well-being, forest bathing was associated with significant improvements in stress reduction, mood enhancement, sleep quality, and relief from insomnia.

### Effects of urban and forest bathing on peripheral blood cell parameters

**Fig. 3:**
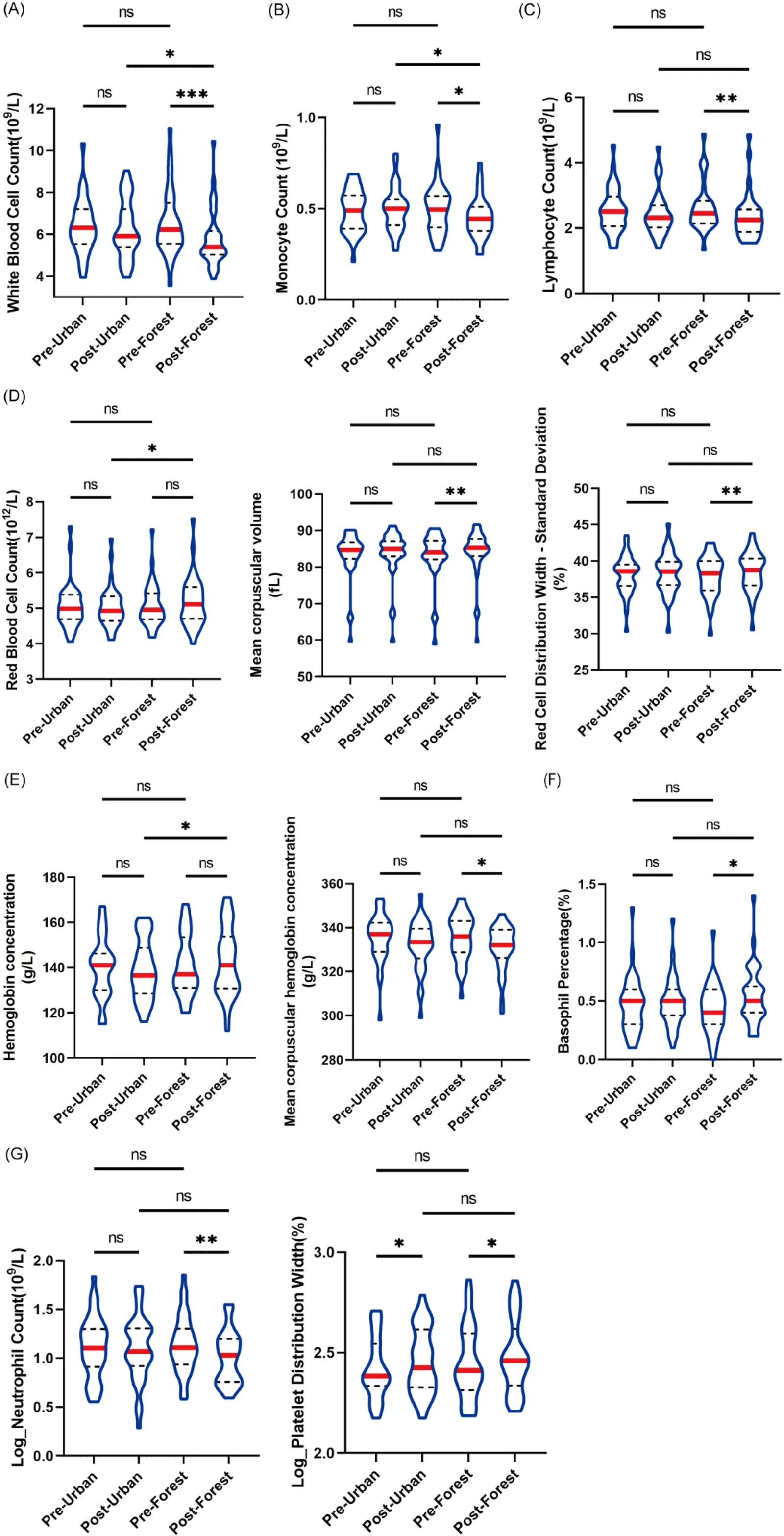
Effects of urban and forest bathing on peripheral blood cell parameters. (A) The total white blood cell counts were significantly reduced after forest bathing (Pre-Forest: 6.5 × 10^9^/L; Post-Forest: 5.8 × 10^9^/L) and were lower than the Post-Urban level (6.3 × 10^9^/L). (B) Monocyte counts decreased significantly following forest bathing (Pre-Forest: 0.50 × 10^9^/L; Post-Forest: 0.45 × 10^9^/L) and were also lower than the Post-Urban level. (C) Lymphocyte counts declined significantly after forest bathing (Pre-Forest: 2.63 × 10^9^/L; Post-Forest: 2.36 × 10^9^/L). (D) Red blood cell counts showed no within-intervention changes, yet the Post-Forest value (5.2 × 10^12^/L) was higher than the Post-Urban value (5.0 × 10^12^/L). (E) Hemoglobin concentration remained unchanged from pre- to post-intervention, but was higher after forest bathing (Post-Forest: 142 g/L) than after urban bathing (Post-Urban: 139 g/L); mean corpuscular hemoglobin concentration decreased following forest bathing (from 336 g/L to 331 g/L). (F) Basophil proportion increased after forest bathing (from 0.43% to 0.54%). (G) Mixed-effects modeling after log-transformation revealed a significant decrease in neutrophil count post-forest bathing and increases in PDW after both urban and forest bathing. Violin plots show individual values (blue), group medians (red dashed lines), and interquartile ranges (black dashed lines) before (“Pre-”) and after (“Post-”) each intervention. *P < 0.05, **P < 0.01, ***P < 0.001; ns, not significant.

The results of our complete blood-count analysis are summarized in Fig. 3. Forest bathing, but not urban bathing, was associated with small but statistically significant shifts in several leukocyte and erythrocyte parameters. Specifically, total white blood cell count decreased from a pre-forest mean of 6.5 × 10^9^/L to 5.8 × 10^9^/L after forest bathing (P < 0.0001), whereas no change was observed before versus after urban bathing (Fig. 3A). Monocyte counts likewise fell significantly following forest bathing (from 0.50 × 10^9^/L to 0.45 × 10^9^/L; P < 0.01) but remained unchanged in the urban setting (Fig. 3B). Lymphocytes declined from 2.63 × 10^9^/L to 2.36 × 10^9^/L after forest bathing (P < 0.01), with only nonsignificant fluctuations seen around urban bathing (Fig. 3C).

In contrast, red blood cell counts did not differ significantly before versus after either intervention; however, post-forest values (5.2 × 10^12^/L) were modestly higher than post-urban values (5.0 × 10^12^/L; P < 0.05), suggesting a transient erythropoietic response to forest exposure (Fig. 3D). This was accompanied by small but significant increases in mean corpuscular volume (from 83 fL to 84 fL; P < 0.01) and red cell distribution width-standard deviation (from 38% to 39%; P < 0.01) after forest bathing. Hemoglobin concentration remained stable across both conditions, yet post-forest levels (142 g/L) were higher than post-urban levels (139 g/L; P < 0.05), while mean corpuscular hemoglobin concentration decreased slightly from 336 g/L to 331 g/L following forest bathing (P < 0.05) (Fig. 3E).

Among the less abundant leukocyte subsets, basophil percentages rose significantly after forest bathing (from 0.43% to 0.54%; P < 0.05) but did not change with urban bathing (Fig. 3F). Neutrophil counts and platelet distribution width (PDW) exhibited right-skewed distributions; after log-transformation and mixed-effects modeling, log_neutrophil counts were found to decrease following forest bathing (P < 0.01), whereas log_PDW increased after both urban and forest bathing (P < 0.05 for each) (Fig. 3G).

Overall, these data indicate that a single session of forest bathing induces modest, short-term reductions in total leukocytes-particularly monocytes, lymphocytes, and neutrophils-alongside slight enhancements in red blood cell indices and basophil proportions.

### Effects of urban and forest bathing on lipid profiles, apolipoproteins, and metabolic markers

**Fig. 4:**
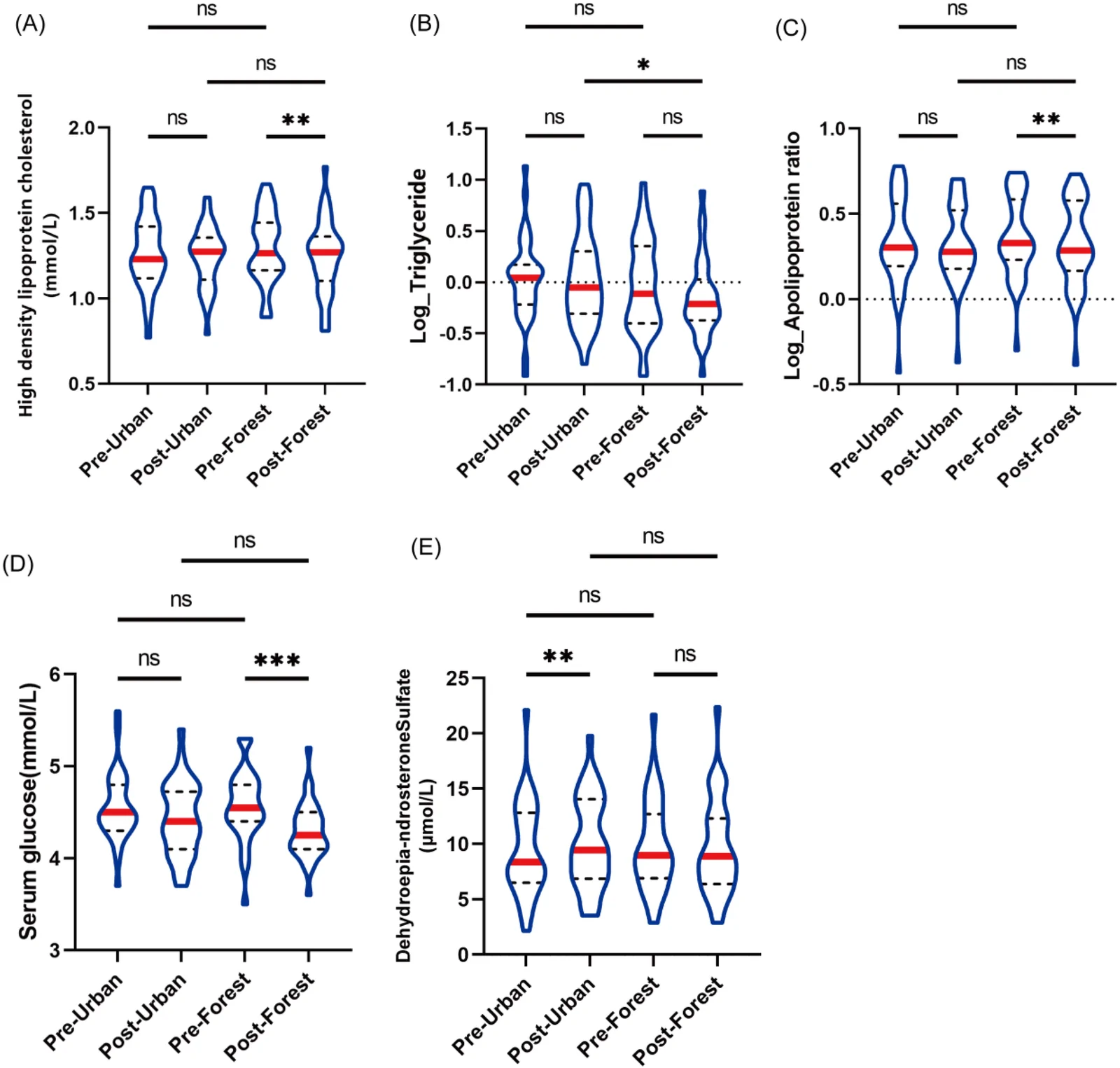
Effects of urban and forest bathing on lipid profiles, apolipoproteins, and metabolic markers. (A) A significant decrease in HDL-C levels following forest bathing (from 1.30 mmol/L to 1.25 mmol/L), suggesting a mild lipid-modulating effect of the forest environment. (B) Post-forest bathing Log-transformed triglycerides (log_triglyceride) levels were significantly lower than Post-urban bathing. (C) A slight decrease in the Log-transformed apolipoprotein ratio (log_apolipoprotein ratio) was observed following forest bathing. (D) Forest bathing significantly reduced blood glucose levels from 4.57 mmol/L to 4.30 mmol/L. (E) DHEA-S levels increased significantly after urban bathing (from 9.44 mmol/L to 10.11 mmol/L), whereas no significant change was observed following forest bathing. Violin plots display individual values (blue), group medians (red dashed lines), and interquartile ranges (black dashed lines) before (“Pre-”) and after (“Post-”) each intervention. *P < 0.05, **P < 0.01, ***P < 0.001; ns, not significant.

HDL-C, a critical cardioprotective factor, remained unchanged after urban bathing but decreased significantly following forest bathing (1.30 mmol/L to 1.25 mmol/L, P < 0.01), suggesting a modest change in HDL-C levels following forest exposure (Fig. 4A). Triglycerides, which serve as the body’s primary energy reservoir, exhibited right-skewed distributions; after log-transformation, log-triglycerides declined more markedly after forest bathing compared to urban bathing (P < 0.05; Fig. 4B). Similarly, the log-transformed apolipoprotein ratio, an important CVD risk indicator, decreased significantly following forest bathing (P < 0.01; Fig. 4C), indicating modest improvement in lipid balance.

Blood glucose levels, a key index of metabolic homeostasis, were unchanged by urban bathing. In contrast, forest bathing induced a clear reduction in average blood glucose (4.57 mmol/L to 4.30 mmol/L, P < 0.0001; Fig. 4D), suggesting a beneficial effect on glycemic control. Meanwhile, DHEA-S, a hormone implicated in stress responses and immune regulation, rose significantly following urban bathing (9.44 mmol/L to 10.11 mmol/L, P < 0.01) but remained stable in the forest condition (Fig. 4E). These findings underscore differential physiological responses to urban versus forest exposures, with forest bathing conferring modest benefits in modulating lipid and glucose metabolism, and stabilizing DHEA-S levels.

## Discussion

In this randomized 2 × 2 crossover trial conducted in a low-latitude subtropical region, we observed that a single session of forest bathing produced significant improvements in psychological well-being and sleep quality, whereas biological changes were generally modest in magnitude. Compared with urban exposure, forest bathing significantly reduced perceived stress and mood disturbance scores and improved subjective sleep quality and insomnia severity. In contrast, hematological, immune, endocrine, and metabolic alterations were selective and remained within physiological ranges. These findings suggest that the most robust short-term effects of forest bathing may occur in psychological domains, with more subtle accompanying biological modulation [29, 46]. Our findings extend this evidence by simultaneously evaluating multiple validated instruments (PSS, POMS, SRSS, and AIS) within a crossover design, thereby strengthening causal inference.

Several mechanisms may underlie these psychological improvements. Forest environments provide multisensory stimuli, including natural visual patterns, phytoncides, natural sounds, and reduced anthropogenic noise, which may promote parasympathetic activation and suppress sympathetic nervous system activity [47–49]. Experimental studies have shown that forest exposure can reduce heart rate, blood pressure, and salivary cortisol, indicating attenuation of hypothalamic–pituitary–adrenal (HPA) axis activation. Reduced stress reactivity may, in turn, facilitate sleep initiation and maintenance, explaining the concurrent improvements in SRSS and AIS scores observed in our study. Collectively, these results support the hypothesis that forest bathing enhances psychological restoration through modulation of autonomic and neuroendocrine stress pathways.

Beyond psychological outcomes, we observed statistically significant but modest reductions in total white blood cell count and specific leukocyte subsets (monocytes, lymphocytes, and neutrophils) following forest bathing. Importantly, all values remained within normal physiological ranges, and the magnitude of change was relatively small. Therefore, these findings should not be interpreted as immune suppression.

Acute psychological stress and sympathetic activation are known to induce transient leukocytosis, particularly involving neutrophils and monocytes, through catecholamine-mediated demargination and redistribution of immune cells [50]. The reductions observed after forest exposure may thus reflect attenuation of stress-related sympathetic arousal and low-grade inflammatory activation rather than diminished immune competence. In this context, forest bathing may promote a more quiescent immune profile characterized by reduced stress-driven leukocyte mobilization.

Although previous forest bathing studies have primarily focused on natural killer (NK) cell activity, our results broaden the scope by examining multiple hematological parameters. The small increase in basophil proportion and subtle changes in erythrocyte indices likely represent short-term physiological adjustments and should be interpreted cautiously. The modest increase in red blood cell indices following forest exposure may reflect acute hemoconcentration related to mild physical exertion or transient shifts in plasma volume rather than true erythropoiesis. Given the short intervention duration, sustained erythropoietic stimulation is unlikely. Overall, the immune-related findings suggest mild immunomodulatory effects consistent with reduced stress burden, rather than clinically significant inflammatory changes.

With respect to metabolic parameters, forest bathing was associated with reductions in blood glucose and log-transformed triglycerides, as well as a modest decrease in the apolipoprotein ratio. These findings align with prior reports indicating that forest walking may improve glycemic control and lipid metabolism [51]. However, changes in HDL-C were small and inconsistent with traditional expectations of cardioprotective improvement, and no significant alterations were observed in total cholesterol or LDL-C. Therefore, the metabolic effects observed here appear subtle and may reflect short-term redistribution or acute energy utilization rather than sustained lipid remodeling.

Notably, serum DHEA-S levels increased significantly following urban exposure but remained stable during forest exposure. DHEA-S is an adrenal steroid hormone regulated by the HPA axis and is often considered a counter-regulatory or stress-responsive hormone [52, 53]. The elevation observed after urban walking may represent a compensatory neuroendocrine response to environmental stressors such as traffic noise, crowding, or air pollution. In contrast, the absence of significant change in the forest condition may indicate reduced HPA axis activation and a more stable endocrine state. These findings support the interpretation that forest environments buffer neuroendocrine stress responses rather than strongly stimulating anabolic hormonal pathways.

Taken together, our findings suggest a coherent pattern: forest bathing produces robust psychological restoration accompanied by modest physiological modulation. It is plausible that improvements in mood and perceived stress precede and partially mediate downstream biological changes. Reduced sympathetic activation and HPA axis activity may contribute to subtle decreases in stress-related leukocyte mobilization and mild improvements in metabolic markers. However, given the short intervention duration, the biological effects are understandably limited in magnitude.

Importantly, although many biological outcomes reached statistical significance, their clinical relevance remains uncertain. Most changes were small and remained within normal ranges. Therefore, forest bathing should currently be regarded as a complementary health-promoting strategy rather than a substitute for established medical interventions.

Because participants walked the same distance and at comparable intensity in both forest and urban settings within a crossover design, the effects of physical activity were balanced between conditions. Nevertheless, we cannot entirely exclude potential synergistic interactions between exercise and environmental context. It is possible that identical physical activity may produce different physiological responses depending on environmental characteristics such as air quality, noise levels, and visual stimuli.

In addition, minute ventilation increases during walking, potentially enhancing inhaled exposure to airborne pollutants. Urban environments may involve higher concentrations of traffic-related pollutants, which could influence stress-related hematological or endocrine responses. Although this reflects real-world exposure differences between environments, future studies incorporating direct environmental monitoring (e.g., particulate matter, noise levels, phytoncide concentrations) would help clarify the relative contributions of environmental quality versus physical activity.

This study has several strengths, including its randomized crossover design, control of sequence and period effects, and comprehensive assessment of psychological, hematological, metabolic, and endocrine parameters. The subtropical low-latitude setting also expands the geographic scope of forest bathing research.

However, several limitations should be acknowledged. First, the sample size was relatively small and comprised healthy young adults, which may limit generalizability. Second, only short-term responses were evaluated; long-term sustainability of these effects remains unknown. Third, mechanistic biomarkers such as inflammatory cytokines, cortisol, or heart rate variability were not directly measured, limiting deeper physiological interpretation. Finally, environmental variables such as air pollutant concentrations were not quantitatively assessed.

## Conclusion

In summary, forest bathing in a subtropical low-latitude region significantly improved psychological well-being and sleep quality and induced modest short-term modulation of immune and metabolic parameters compared with urban exposure. The findings support the role of forest environments as a complementary, non-pharmacological strategy for stress reduction and health promotion. Further large-scale and mechanistic studies are warranted to clarify long-term clinical significance and underlying biological pathways.

## Supplementary information

**Additional file 1:** **Supplementary Table S1.** Standard items for inclusion and exclusion. **Supplementary Table S2.** Baseline demographic characteristics and clinical parameters of participants by group. **Supplementary Table S3.** Paired-sample t-test results comparing baseline measurements before Period 1 and Period 2 to assess washout period adequacy. **Supplementary Table S4.** Mixed-effects model analysis examining Period × Sequence interactions to assess potential carryover effects.

## Declarations

### Adverse events

There were no serious adverse events (unplanned hospitalization or death) in this trial. Participant A completed only one urban bath and towards the end of the week of the washout period was suddenly informed that a family member was ill and needed to be cared for and was unable to continue to complete the forest bath. Participant B withdrew from the trial at the start of the trial without informing the exact reason.

### Data availability statement

The raw data supporting the conclusions of this study are publicly available. The dataset is accessible in the [https://figshare.com/s/17f841b6ea099fca84ef] repository maintained by the figshare. The repository provides detailed documentation and instructions for accessing and using the data.
