# Supplementary material for: Short-term effects of urban versus forest bathing on stress, mood, sleep, immune function, and metabolic health: a randomized, open-label, 2 × 2 crossover trial
Source: Environ Health Prev Med. 2026 Aug 27;31:55. doi: 10.1265/ehpm.26-00092 (PMC13537900; doi:10.1265/ehpm.26-00092)
Supplement: Supplementary file 1 [file ehpm-31-055-s001.docx]

Supplementary Table S1. Standard items for inclusion and exclusion

| Serial Number | Inclusion criteria | Exclusion Criteria |
| --- | --- | --- |
| (1) | Healthy subjects, male or female, aged 18-45, never smoking, in good health. | Participants who participated in any drug clinical trials within 3 months prior to the trial. |
| (2) | Body mass index 19-26, male weight ≥ 50 kg, female weight ≥ 45 kg. | Individuals with chronic or active gastrointestinal diseases such as esophageal disease, gastritis, gastric ulcer, enteritis, active gastrointestinal bleeding, or gastrointestinal surgery within three years. |
| (3) | Fully understand and voluntarily sign the informed consent form. | Individuals with a history of cardiovascular system, endocrine system, urinary system, nervous system, respiratory system, hematology, immunology (including personal or family history of inherited immune deficiency), psychiatry, metabolic abnormalities, etc., who are still clinically significant according to researchers. |
| (4) |  | Individuals with a history of allergies to drugs, food, or other substances. |
| (5) |  | Individuals with a history of cancer, epilepsy, frequent headaches, and tuberculosis infection. |
| (6) |  | Those who cannot tolerate venous puncture or have a history of dizziness or needle fainting. |
| (7) |  | Patients who have undergone surgery within 6 months prior to the experiment, which has been determined by the researchers to affect drug absorption, distribution, metabolism, and excretion; Or have undergone surgical procedures within 3 months prior to the experiment; Or those who plan to undergo surgical procedures during the study period. |
| (8) |  | Those who received the vaccine within 3 months before the experiment. |
| (9) |  | Individuals who have used any medication within the 30 days prior to the trial. |
| (10) |  | Blood donors or those who have lost a large amount of blood (>400 mL) within 3 months before the experiment. |
| (11) |  | Alcoholics or frequent drinkers within the 6 months prior to the experiment, who consume more than 14 units of alcohol per week (1 unit=360 mL of beer or 45 mL of 40% spirits or 150 mL of wine); Those who are unwilling to stop drinking alcohol or using any alcoholic products during the trial period; Or the alcohol breath test result is positive (>0.0 mg/100 mL) |
| (12) |  | Individuals who consume excessive amounts of tea, coffee, and/or caffeinated beverages (8 or more cups, 1 cup=250 mL) daily, or who do not agree to stop drinking tea, coffee, and/or caffeinated beverages during the trial period. |
| (13) |  | Individuals who consume a diet that may affect the metabolism of the investigational drug within 7 days prior to taking the drug (including grapefruit or grapefruit products, oranges, oranges, strawberries, apricots, xanthine diet, etc.), or who the researcher believes have other diets that may affect the absorption, distribution, metabolism, and excretion of the drug, or who do not agree to stop consuming the aforementioned diet during the trial period. |
| (14) |  | Individuals who have special dietary requirements and cannot follow a uniform diet, or those who are lactose intolerant. |
| (15) |  | Female participants who engage in unprotected sexual activity within 2 weeks prior to the trial, or who have a pregnancy plan, sperm or egg donation plan within 6 months from screening to the end of the trial, or who refuse to use one or more non pharmacological contraceptive measures (such as complete abstinence, contraceptive rings, partner ligation, etc.) during the trial period. |
| (16) |  | Drug abusers or those who have used soft drugs (such as marijuana) within the past 3 months prior to the experiment, or those who have used hard drugs (such as cocaine, phencyclidine, etc.) within the past 1 year prior to the experiment. |
| (17) |  | For patients with creatinine clearance rate<80 mL/min, the Cockcroft Gault formula^[1]^ for calculating creatinine clearance rate is CrCl=[(140-age) x body weight (kg)]/[0.814 x Scr (µmol/L)]. When calculating creatinine clearance rate, attention should be paid to the unit of creatinine; Women are calculated to be 0.85. |
| (18) |  | Individuals with positive results in urine drug screening (morphine, methamphetamine, ketamine, methylenedioxymethamphetamine, tetrahydrocannabinol acid, cocaine). |
| (19) |  | Individuals with clinically significant abnormalities in physical examination, electrocardiogram, laboratory tests, color ultrasound, chest radiograph, vital signs, and laboratory related examinations (subject to clinical judgment). |
| (20) |  | Individuals with positive screening results for infectious diseases. |
| (21) |  | Individuals with positive results in nicotine urine testing. |
| (22) |  | Female participants who have used oral contraceptives within 30 days prior to the trial. |
| (23) |  | Female subjects who have used long-acting estrogen or progesterone injections or implants within 6 months prior to the trial. |
| (24) |  | Pregnant or lactating women, or those whose blood pregnancy test is positive. |
| (25) |  | Participants may not be able to complete this study due to other reasons or may have other reasons deemed inappropriate by the researchers to participate in the trial. |

Reference

1. Cockcroft DW, Gault MH. Prediction of creatinine clearance from serum creatinine. Nephron. 1976;16(1):31-41. doi: 10.1159/000180580. PMID: 1244564.

Supplementary Table S2. Baseline demographic characteristics and clinical parameters of participants by group

| Variable | Group A | Group B | P-value^a^ |
| --- | --- | --- | --- |
| Sex, n  Females  males | 8  10 | 12  4 | 0.09 |
| Age (y) | 24.94±5.87 | 23.81±4.04 | 0.66 |
| Athens Insomnia Scale (AIS) | 5.89±3.16 | 6.88±3.07 | 0.92 |
| Self-Rating Scale of Sleep (SRSS) | 21.22±4.29 | 21.56±3.83 | 0.66 |
| Profile of Mood States (POMS) | 38.28±13.26 | 50.44±22.51 | 0.33^b^ |
| Perceived Stress Scale (PSS) | 23.44±5.55 | 26.56±5.83 | 0.14^b^ |
| White blood cell count (10^9^/L) | 6.50±1.82 | 6.14±1.21 | 0.51 |
| Monocyte count (10^9^/L) | 0.50±0.17 | 0.52±0.12 | 0.80^b^ |
| Percentage of monocytes (%) | 7.52±1.81 | 8.66±2.61 | 0.60^b^ |
| Neutrophil count (10^9^/L) | 3.17±1.07 | 2.89±0.94 | 0.42 |
| Red blood cell count (10^12^/L) | 4.92±0.48 | 4.87±0.44 | 0.76 |
| Lymphocyte count (10^9^/L) | 2.68±0.87 | 2.58±0.55 | 0.43 |
| Lymphocyte percentage (%) | 39.92±5.64 | 42.37±7.05 | 0.27 |
| Mean corpuscular volume (fL) | 82.03±7.79 | 84.04±5.71 | 0.70^b^ |
| Red cell distribution width-standard deviation (%) | 37.43±3.04 | 38.38±2.00 | 0.30^b^ |
| Red cell distribution width-coefficient of variation (%) | 12.82±1.31 | 12.63±1.07 | 0.98^b^ |
| Mean red cell hemoglobin content (pg) | 27.52±3.14 | 28.26±2.32 | 0.77 |
| Hemoglobin concentration (g/L) | 141.56±12.89 | 137.25±13.49 | 0.35 |
| Mean corpuscular hemoglobin concentration (g/L) | 334.72±11.17 | 335.94±8.35 | 0.72 |
| Basophil count (10^9^/L) | 0.03±0.01 | 0.02±0.01 | 0.97^b^ |
| Basophil percentage (%) | 0.43±0.20 | 0.42±0.22 | 0.90 |
| Eosinophil count (10^9^/L) | 0.15±0.07 | 0.13±0.05 | 0.47 |
| Eosinophil percentage (%) | 2.18±1.11 | 2.18±0.82 | 0.40^b^ |
| Log_neutrophil count | 1.10±0.33 | 1.01±0.32 | 0.43 |
| Neutrophil percentage (%) | 49.93±5.62 | 46.37±7.90 | 0.14 |
| Log_platelet distribution width | 2.44±0.17 | 2.40±0.16 | 0.50 |
| Platelet count (10^9^/L) | 257.44±47.95 | 261.56±59.93 | 0.83 |
| Mean platelet volume (fL) | 10.19±0.95 | 10.12±0.96 | 0.99^b^ |
| Thrombocytocrit(%) | 0.26±0.05 | 0.26±0.05 | 0.98 |
| C-reactive protein (CRP) (mg/L) | 2.74±0.86 | 3.11±1.83 | 0.91^b^ |
| High density lipoprotein cholesterol (HDL-C) (mmol/L) | 1.29±0.23 | 1.25±0.19 | 0.54 |
| Low density lipoprotein cholesterol (LDL-C) (mmol/L) | 2.72±0.46 | 2.71±0.48 | 0.92 |
| Log_triglyceride | -0.06±0.42 | 0.09±0.49 | 0.35 |
| Apolipoprotein A1 (Apo A1) (g/L) | 1.19±0.15 | 1.17±0.18 | 0.75 |
| Apolipoprotein B (Apo B) (g/L) | 0.81±0.14 | 0.85±0.16 | 0.50 |
| Lipoprotein(a) [Lp(a)] (g/L) | 141±156.83 | 243.19±246.49 | 0.15^b^ |
| Total cholesterol (mmol/L) | 4.59±0.63 | 4.68±0.67 | 0.70 |
| Log_apolipoprotein ratio | 0.39±0.21 | 0.33±0.29 | 0.66^b^ |
| Brain natriuretic peptide (BNP) (ng/L) | 54.62±31.70 | 57.88±48.17 | 0.41^b^ |
| Serum glucose (mmol/L) | 4.49±0.40 | 4.52±0.34 | 0.82 |
| Insulin (μU/mL) | 14.09±10.72 | 10.15±4.46 | 0.80^b^ |
| Dehydroepiandrosterone sulfate (DHEA-S) (μmol/L) | 9.74±4.90 | 9.52±3.18 | 0.88 |

*Note*: Comparison of Group A and Group B factors all showed P > 0.05, P>0.05 indicates that there is insufficient statistical evidence to prove that there is a difference between the two groups in these indicators, and the grouping is balanced and comparable.

P-value^a^ derived from unpaired t-tests between the two groups if both groups met the normality test, unless otherwise stated.

P-value^b^ derived from Kolmogorov-Smirnov test of means between two groups if both groups did not meet the normality test.

**Supplementary Table S3.** Paired-sample t-test results comparing baseline measurements before Period 1 and Period 2 to assess washout period adequacy.

| Variable | Mean difference | T-value | P-value | **95% CI** |
| --- | --- | --- | --- | --- |
| AIS | 0.71 | 1.92 | 0.063 | [-0.04, 1.45] |
| SRSS | 1.85 | 2.96 | 0.016^*^ | [0.58, 3.13] |
| POMS | 5.47 | 2.38 | 0.023^*^ | [0.79, 10.15] |
| PSS | 0.24 | 0.38 | 0.708 | [-1.03, 1.50] |
| White blood cell count (10^9^/L) | -0.15 | -0.77 | 0.449 | [-0.54, 0.25] |
| Monocyte count (10^9^/L) | -0.01 | -0.52 | 0.607 | [-0.07, 0.04] |
| Percentage of monocytes (%) | 0.48 | 1.24 | 0.223 | [-0.31, 1.27] |
| Neutrophil count (10^9^/L) | -0.10 | -0.78 | 0.439 | [-0.37, 0.17] |
| Red blood cell count (10^12^/L) | 0.02 | 0.50 | 0.619 | [-0.06, 0.10] |
| Lymphocyte count (10^9^/L) | -0.03 | -0.24 | 0.811 | [-0.29, 0.23] |
| Lymphocyte percentage (%) | 1.68 | 1.47 | 0.150 | [-0.64, 4.01] |
| Mean corpuscular volume (fL) | -0.37 | -2.40 | 0.022^*^ | [-0.69, -0.06] |
| Red cell distribution width-standard deviation (%) | -0.23 | -1.55 | 0.130 | [-0.53, 0.07] |
| Red cell distribution width-coefficient of variation (%) | -0.02 | -0.50 | 0.619 | [-0.09, 0.05] |
| Mean red cell hemoglobin content (pg) | -0.16 | -2.30 | 0.028^*^ | [-0.31, -0.02] |
| Hemoglobin concentration (g/L) | -1.91 | -1.86 | 0.072 | [-4.00, 0.18] |
| Mean corpuscular hemoglobin concentration (g/L) | -0.35 | -0.27 | 0.786 | [-2.97, 2.27] |
| Basophil count (10^9^/L) | -0.00 | -0.65 | 0.523 | [-0.01, 0.00] |
| Basophil percentage (%) | -0.03 | -0.84 | 0.409 | [-0.09, 0.04] |
| Eosinophil count (10^9^/L) | 0.01 | 0.93 | 0.361 | [-0.01, 0.02] |
| Eosinophil percentage (%) | 0.02 | 0.20 | 0.841 | [-0.16, 0.20] |
| Log_neutrophil count | -0.10 | -0.78 | 0.439 | [-0.37, 0.17] |
| Neutrophil percentage (%) | -2.16 | -1.69 | 0.100 | [-4.75, 0.44] |
| Log_platelet distribution width | -0.03 | -2.25 | 0.031^*^ | [-0.06, -0.00] |
| Platelet count (10^9^/L) | 2.06 | 0.78 | 0.442 | [-3.32, 7.44] |
| Mean platelet volume (fL) | -0.09 | -1.56 | 0.127 | [-0.22, 0.03] |
| Thrombocytocrit (%) | -0.00 | -0.11 | 0.912 | [-0.01, 0.01] |
| CRP (mg/L) | 0.41 | 1.24 | 0.225 | [-0.26, 1.08] |
| HDL-C (mmol/L) | -0.01 | -0.64 | 0.529 | [-0.06, 0.03] |
| LDL-C (mmol/L) | 0.12 | 2.53 | 0.016^*^ | [0.02, 0.22] |
| Log_triglyceride | 0.02 | 0.37 | 0.711 | [-0.07, 0.11] |
| Apo A1 (g/L) | -0.01 | -0.57 | 0.574 | [-0.05,0.03] |
| Apo B (g/L) | -0.02 | -1.98 | 0.056 | [-0.04, 0.00] |
| Lp(a) (g/L) | 2.85 | 0.78 | 0.442 | [-4.61, 10.32] |
| Total cholesterol (mmol/L) | -0.00 | -0.06 | 0.951 | [-0.16, 0.15] |
| Log_apolipoprotein ratio | 0.03 | 2.14 | 0.040^*^ | [0.00, 0.06] |
| BNP (ng/L) | 8.97 | 1.12 | 0.269 | [-7.26, 25.20] |
| Serum glucose (mmol/L) | 0.05 | 0.94 | 0.356 | [-0.06, 0.16] |
| Insulin (μU/mL) | 0.25 | 0.20 | 0.841 | [-2.23, 2.72] |
| DHEA-S (μmol/L) | -0.02 | -0.11 | 0.915 | [-0.49, 0.44] |

*Note*: Mean difference: The average of the baseline value in the second phase minus the baseline value in the first phase, representing the overall difference magnitude between the two baselines; T - value: The test statistic of the paired t - test, reflecting the degree of difference between two sets of paired data; P - value: The level of statistical significance, the core basis for judgment; 95% CI (95% confidence interval): The 95% confidence interval of the mean difference. If the interval contains 0, it is equivalent to P>0.05, further corroborating that the washout period is sufficient. Although several variables showed statistically significant differences between baseline measurements, the overall pattern did not indicate systematic residual effects from the preceding intervention.

**Supplementary Table S4.** Mixed-effects model analysis examining Period × Sequence interactions to assess potential carryover effects.

| Variable | Mean difference | T-value | P-value |
| --- | --- | --- | --- |
| AIS | 0.63 | 0.85 | 0.404 |
| SRSS | -0.16 | -0.13 | 0.901 |
| POMS | -4.19 | -0.91 | 0.371 |
| PSS | 0.92 | 0.73 | 0.470 |
| White blood cell count (10^9^/L) | 0.36 | 0.93 | 0.361 |
| Monocyte count (10^9^/L) | 0.02 | 0.42 | 0.680 |
| Percentage of monocytes (%) | -0.32 | -0.40 | 0.691 |
| Neutrophil count (10^9^/L) | 0.16 | 0.60 | 0.554 |
| Red blood cell count (10^12^/L) | 0.02 | 0.28 | 0.780 |
| Lymphocyte count (10^9^/L) | 0.17 | 0.66 | 0.512 |
| Lymphocyte percentage (%) | 0.29 | 0.12 | 0.903 |
| Mean corpuscular volume (fL) | -0.15 | -0.46 | 0.647 |
| Red cell distribution width-standard deviation (%) | -0.17 | -0.58 | 0.566 |
| Red cell distribution width-coefficient of variation (%) | -0.06 | -0.80 | 0.428 |
| Mean red cell hemoglobin content (pg) | -0.02 | -0.11 | 0.913 |
| Hemoglobin concentration (g/L) | 1.58 | 0.76 | 0.451 |
| Mean corpuscular hemoglobin concentration (g/L) | 0.63 | 0.24 | 0.811 |
| Basophil count (10^9^/L) | -0.00 | -0.09 | 0.929 |
| Basophil percentage (%) | -0.06 | -0.97 | 0.338 |
| Eosinophil count (10^9^/L) | 0.00 | 0.35 | 0.726 |
| Eosinophil percentage (%) | -0.14 | -0.81 | 0.422 |
| Log_neutrophil count | 0.16 | 0.60 | 0.554 |
| Neutrophil percentage (%) | 0.24 | 0.09 | 0.928 |
| Log_platelet distribution width | 0.05 | 2.03 | 0.051 |
| Platelet count (10^9^/L) | -5.91 | -1.12 | 0.271 |
| Mean platelet volume (fL) | 0.21 | 1.82 | 0.078 |
| Thrombocytocrit (%) | 0.00 | 0.33 | 0.740 |
| CRP (mg/L) | -0.59 | -1.03 | 0.309 |
| HDL-C (mmol/L) | 0.07 | 1.59 | 0.123 |
| LDL-C (mmol/L) | -0.00 | -0.05 | 0.965 |
| Log_triglyceride | -0.14 | -1.62 | 0.114 |
| Apo A1 (g/L) | 0.05 | 1.39 | 0.174 |
| Apo B (g/L) | -0.01 | -0.67 | 0.507 |
| Lp(a) (g/L) | 2.56 | 0.34 | 0.734 |
| Total cholesterol (mmol/L) | 0.35 | 2.45 | 0.020^*^ |
| Log_apolipoprotein ratio | 0.06 | 2.56 | 0.015^*^ |
| BNP (ng/L) | 8.33 | 0.52 | 0.610 |
| Serum glucose (mmol/L) | 0.04 | 0.33 | 0.746 |
| Insulin (μU/mL) | 2.37 | 0.97 | 0.339 |
| DHEA-S (μmol/L) | 0.82 | 1.85 | 0.074 |

*Note:* Mixed-effects models were fitted using the lmer package in R with the model: *Outcome ~ Period × Sequence + (1 | Participant)*. Except for total cholesterol and the log-transformed apolipoprotein ratio, no significant Period × Sequence interactions were detected (*P* > 0.05), supporting the adequacy of the 7-day washout period.
